# Predicting visual field boundaries from head features

**DOI:** 10.1364/JOSAA.551858

**Published:** 2025-05-19

**Authors:** Uday Nakade, Manuel Spitschan

**Affiliations:** 1 Max Planck Institute for Biological Cybernetics, Max Planck Research Group Translational Sensory & Circadian Neuroscience, Tübingen, Germany; 2 Technical University of Munich, TUM School of Medicine and Health, Chronobiology & Health, Munich, Germany; 3 Technical University of Munich, TUM Institute for Advanced Study (TUM-IAS), Garching, Germany

## Abstract

Light exposure profoundly affects human physiology, including circadian rhythms and hormonal regulation. Current methods to assess light exposure often ignore anatomical factors that influence how much light reaches the retina. This study introduces a novel simulation pipeline to model visual field (VF) boundaries as a function of head anatomy. Using the parametric ICT Face Model and the Mitsuba 3 rendering engine, we generated diverse 3D head shapes and simulated light interactions to predict VF boundaries. The results reveal significant variability in VF boundaries, influenced by anatomical features such as the nose, cheeks, and eyebrows. This leads to differences in projected solid angles of the VF of up to 18.7%. This study highlights the importance of individual approaches in estimating light exposure.

## 1. BACKGROUND

Beyond vision, light exposure strongly influences human physiology by synchronizing the internal clock to environmental time and modifying the production of the hormone melatonin [1–3]. These “non-visual” effects of light are mediated by a set of retinal ganglion cells expressing the short-wavelength-sensitive photopigment melanopsin [4], which, along with the cones and rods, encodes ambient light intensity. In 2018, the International Commission on Illumination (CIE) standardized the spectral sensitivity of human photoreceptors, introducing novel quantities for measuring and quantifying the “non-visual” effects of light [5,6].

Conventionally, light exposure is measured using spectral irradiance or photopic illuminance measurements in the corneal plane, including using wearable light loggers [7,8]. The amount of light that reaches the retina from environmental exposure depends on several factors that are not considered in corneal irradiance measurements [9,10]. First, the area of the eye’s aperture, the pupil, modifies retinal irradiance [11] and impacts the physiological effects of light [12,13]. Second, due to self-obstruction from head shape, the effective human visual field (VF) or field of view is not 90° in all directions (hemispherical) but spans from 60° toward the nose to approximately 107° toward the temple and from 60° upward to 70° downward. The estimates vary with measurement techniques [14]. CIE S 026:2018 [6] recommends that the field of view should be limited to 50° upward and 70° downward when considering binocular vision. Since most of the lighting in residential and office spaces is placed on the ceiling, blockage from the eyebrow region can lead to significant differences compared to irradiance measurements. The magnitude of this misestimation depends on the overall scene geometry [15].

The effects of a limited VF can extend beyond light exposure. Reference [16] shows that individual differences in VFs can explain the advantage of the lower visual field in crowding scenarios. To ensure that peripheral visual field defects are not missed due to facial anatomy in routine perimetry, Ref. [17] recommends turning the head to increase the coverage of angles.

Computer rendering has made significant contributions to lighting design and virtual reality. Knowledge of human perception has led to advances in rendering techniques [18,19]. In this paper, we intend to demonstrate the utility of physically realistic rendering techniques in vision science.

Here, we investigate the role of head shape in limiting the effective VF in humans through a novel simulation pipeline incorporating a parametric 3D head shape model and spectral rendering. We demonstrate that, using numeric simulation techniques, we can parametrically describe head shapes and estimate the variability of VF depending on the head shape.

## 2. OVERVIEW OF THE PIPELINE

**Fig. 1.**
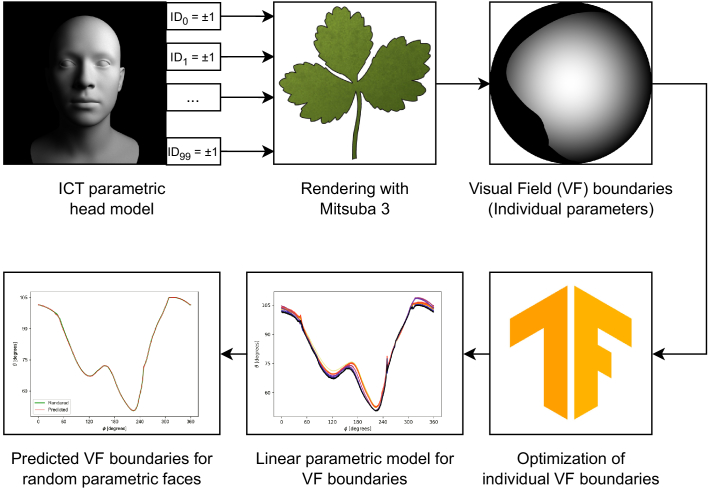
Overview of the approach taken to predict VF boundaries for random faces.

In brief, to study the effect of head anatomy on the VF, we first deploy a parametric model for faces to generate variability in head shapes. We use the physically realistic rendering engine Mitsuba 3 to generate radiance images from the point of view of the eye. We can map the effect of individual parameters on the VF to create a linear model and predict the VF for novel head shapes. Figure 1 shows an overview of the approach taken in this paper. All code used in this work is available under the MIT License on GitHub [20] and archived in Zenodo [21].

### A. Toolbox

#### 1. Generating Parametric Head Shapes Using a Benchmark Face Model

**Fig. 2.**
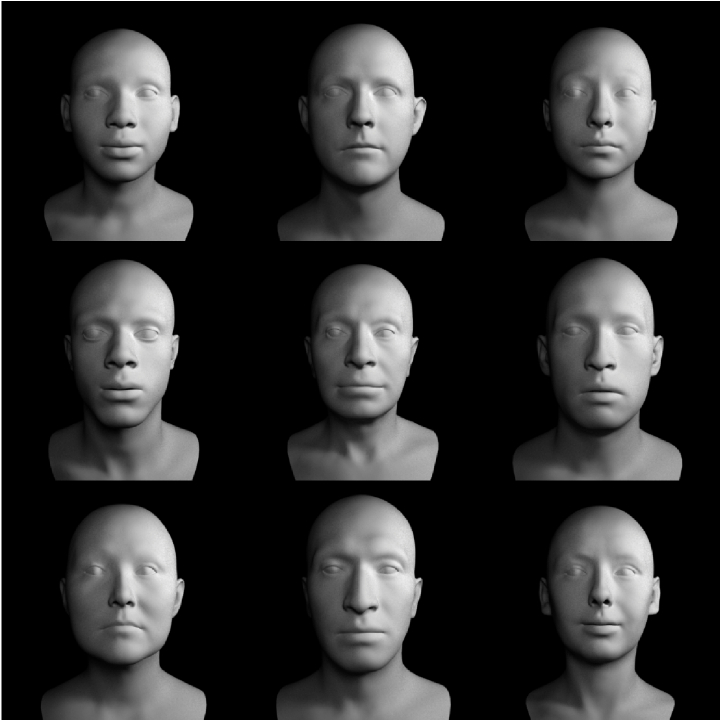
Exemplary faces created using the ICT model.

The ICT Face Model [22] is a morphable, parametric model for face identities and expressions. The model was created by scanning the faces of 79 individuals (34 female and 45 male) using a 25-camera setup, which allowed for pore-level accuracy. To increase the diversity in the dataset, the ICT Face Model also includes 99 face scans from Triplegangers [23]. It is a 3D mesh with 26,719 vertices and 26,384 facets. Starting from a generic neutral mesh ( $M_{g}$ ), the model provides $N_{IDs}=100$ principal component analysis (PCA) morph targets in the facial identity space. All the model’s vertices can be linearly and continuously transformed along these PCA directions to generate novel faces using a technique called blendshapes [24]. The mathematical equation driving this is

(1) $$M_{newface}=M_{g}+\sum_{i=0}^{N_{IDs}-1}c_{i}\times \left(M_{i}-M_{g}\right),$$

where $c_{i}$ is the component along the i th PCA direction. Figure 2 shows exemplary faces created using this model.

#### 2. Physically Realistic Spectral Rendering

To get the VF, we decided not to calculate it using the geometric description of the face meshes but to place a camera at the location of the eyes and render an image of the surroundings using the scene description. We needed a physically realistic rendering engine and chose Mitsuba 3 [25], which is a cutting-edge light transport simulation and rendering framework, leveraging Monte Carlo methods designed for efficient CPU and GPU usage. As rendering is an ideal way of parametrically changing scene aspects in experiments without creating real-world setups, Mitsuba 3 and its earlier versions have been used previously to generate stimuli in vision science [26–28].

**Fig. 3.**
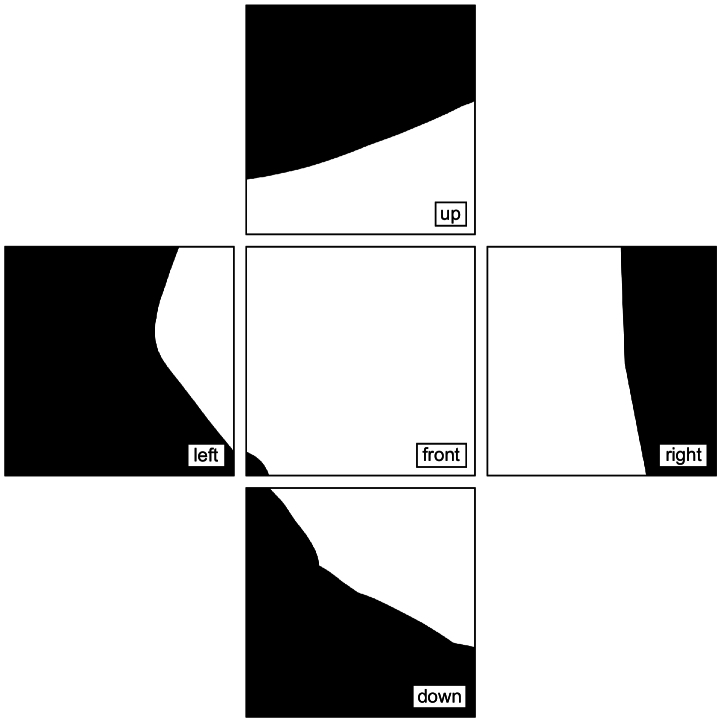
Images obtained from Mitsuba 3 by placing the camera at the eye of the generic head model and pointing it in various directions. The five sensors lie on a cube, which has been “unwrapped” to create this illustration.

**Fig. 4.**
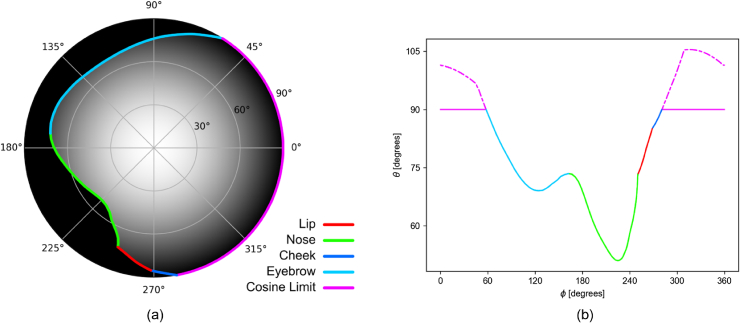
Visual field representations for the generic head model. (a) The visual field represented as an image. θ
 is in the radial direction, and ϕ
 is in the angular direction. (b) The visual field boundary with θ
 plotted against ϕ
 .

### B. *In Silico* Experimental Setup

Along with the generic neutral face shape (all identity parameters = 0), the faces corresponding to each of the $N_{IDs}=100$ PCA ID parameters being +1 and −
1 were chosen, while all the other parameters were kept 0. This resulted in a total of 201 face shapes. A constant radiance light source surrounded the head from all sides. A perspective camera was placed just outside the right eye (at a distance of less than $2\times 10^{-4}mm$ ). The camera’s field of view (FOV) was 90°, and the resolution was 1024×
1024 . Five images were rendered to cover the entire hemisphere in front of the eye by pointing the camera in various directions (the camera setup is described in Appendix B). The head model itself was made entirely non-reflective. This created complete contrast in the rendered images between the directions blocked by the facial anatomy and those that were not, facilitating locating the pixels corresponding to the VF boundary.

### C. Obtaining the Visual Field from Rendered Images

Mitsuba gave five radiance images per face shape (example output shown in Fig. 3). The value at each pixel was the radiance from the direction joining the pixel and the eye. A polar coordinate system was used to denote these directions, with θ
 being measured away from the ray pointing directly in front of the eye and ϕ
 measuring the angle around it (counter-clockwise starting from the direction pointing horizontally away from the nose). Each pixel in the images that lay on the boundary between the occlusions and the constant light source (VF boundary) gave a pair of (θ
,ϕ
) coordinates. The 360° range of ϕ
 was split into 36,000 equally spaced bins. The θ
 values of all the pixels in each bin were averaged to get one θ
 per ϕ
 bin, thus giving a function θ
(ϕ
) (see Appendix C).

### D. Effect of Individual ID Parameters on the Visual Field

**Fig. 5.**
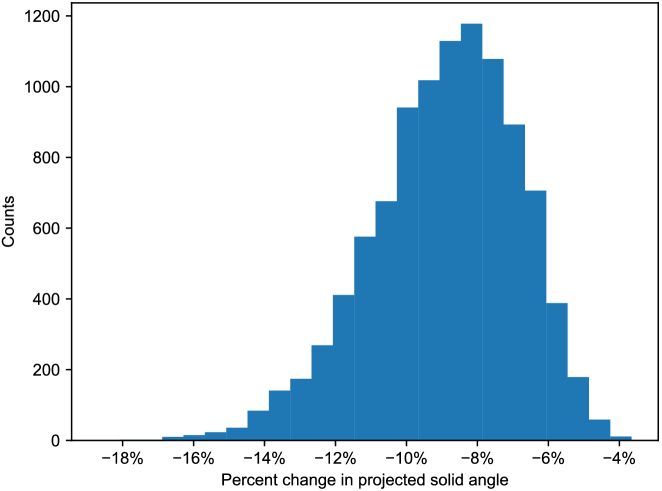
Histogram of the percentage change in the projected solid angle of the visual field as compared to a cosine-weighted hemisphere, for 10,000 random faces.

Figure 4 shows the VF and its boundary for the generic neutral face (all PCA ID parameters = 0). The projected solid angle calculated using this VF was 2.8277 sr, which represents a decrease of 10% from the projected solid angle of a hemisphere ( π
sr ). We generated 10,000 random faces by uniformly sampling $c_{i}$ s between −
1 and 1. Figure 5 shows a histogram of the decrease in the projected solid angle. The values range from 3.65% to 18.7%, with the mean and median being 8.92% and 8.73%, respectively. This shows how important accounting for the head shape can be.

**Fig. 6.**
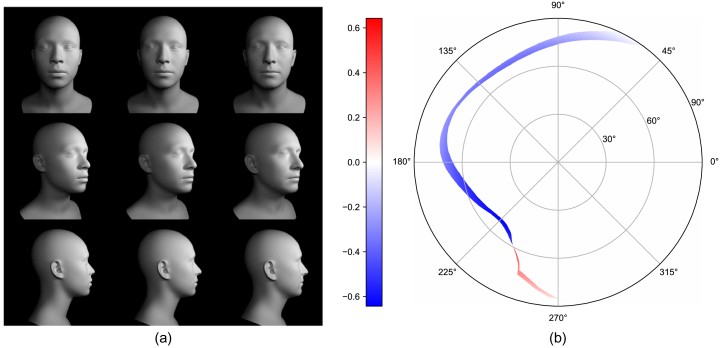
(a) Change in the face shape when ID parameter number 2 goes from −
1 to 0 to +1 (left to right). (b) Corresponding change in the visual field. This leads to a difference of 2.16% in the projected solid angle.

Figure 6 shows the change in the VF when the ID parameter number 2 is changed from −
1 to 1. Please refer to Refs. [20,21] for the individual effects of other ID parameters.

## 3. PREDICTING VISUAL FIELDS FROM PCA COMPONENTS OF IDENTITY

Similar to calculating the mesh for a new face (described by PCA components $c_{i}$ ) as a linear combination of the meshes corresponding to the individual parameters (Eq. (1)), it is possible to do the same for VF boundaries. This is a non-linear calculation, which we have approximated with a linear model.

Let $\theta_{\pm i}(\phi )$ be the VF boundary when the i th PCA parameter is set to ±
1 , with i going from 0 to $N_{IDs}-1=99$ . Let θ
g(ϕ
) be the VF boundary for the generic neutral face. Now, for a randomly generated face with PCA components $c_{i}$ , the VF boundary can be predicted by linearly combining the deviations θ
+i−
θ
g as follows:

(2) θ
random=θ
g+∑
i=0NIDs−
1ci⋅
(θ
+i−
θ
g).

As a second approximation, we treated the +ve and −
ve directions of each PCA component as separate parameters, which got us the following equation for the VF boundaries of random faces:

(3) θ
random=θ
g+∑
i=0NIDs−
1|ci|⋅
(θ
sign(ci)i−
θ
g).

This method suggests that if a person’s head shape can be expressed as linear weights in the ICT model, predicting VF boundaries using Eq. (3) would be possible. This remains to be demonstrated empirically.

## 4. OPTIMIZATION OF THE VISUAL FIELD BOUNDARY FOR LINEAR INTERPOLATION

**Fig. 7.**
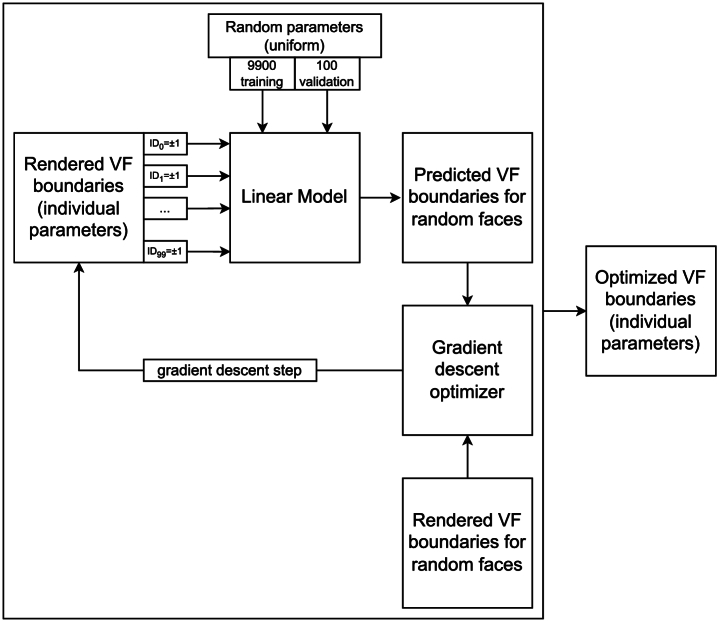
Flow diagram showing the optimization process for the visual field boundaries of faces corresponding to the generic model and the individual PCA parameters.

As we got VF boundaries for individual parameters using the rendering output and as the pixels were discrete, the VF boundaries were not exactly smooth. To minimize the error between predicted and rendered VF boundaries for random head shapes, we decided to optimize $\theta_{\pm i}(\phi )$ and θ
g(ϕ
) using TensorFlow. Figure 7 shows a flowchart of the process. $N_{random+validation}=10,000$ random head shapes were generated, of which, $N_{validation}=100$ were reserved for validation. PCA parameters $c_{i}$ were uniformly sampled between (−
1,1) . We chose the following as the loss function:

(4) L=0.75⋅
∑
ϕ
∑
j=0Nrandom−
1(θ
randomj,predicted−
θ
randomj,rendered)2+0.25⋅
∑
ϕ
((θ
g,optimized−
θ
g,rendered)2+∑
i=0NIDs−
1∑
±
(θ
±
i,optimized−
θ
±
i,rendered)2).

The first term measures the squared error of the predicted VF boundaries for random head shapes compared to the rendered ones. In contrast, the second term ensures that the optimized VF boundaries for the generic neutral head shape and the individual parameters will not veer too far off from their rendered values. The factors in the front (0.75 and 0.25) ensure that more weight is given to the predictions for the random head shapes. The factors themselves were selected arbitrarily, except that they summed to one. With a learning rate $lr=5\times 10^{-8}$ , a simple gradient descent algorithm was followed in TensorFlow until the validation loss, which is given by

(5) Lvalidation=∑
ϕ
∑
j=0Nvalidation−
1(θ
validationj,predicted−
θ
validationj,rendered)2,

stopped decreasing.

**Fig. 8.**
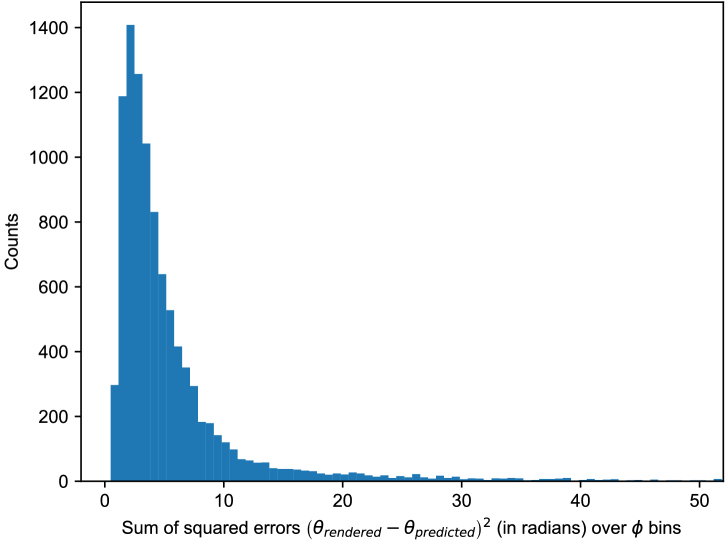
Histogram of squared errors in the predicted boundaries of 10,000 random faces. The x axis has been truncated for visibility, as 99% of the errors are below 50.

**Fig. 9.**
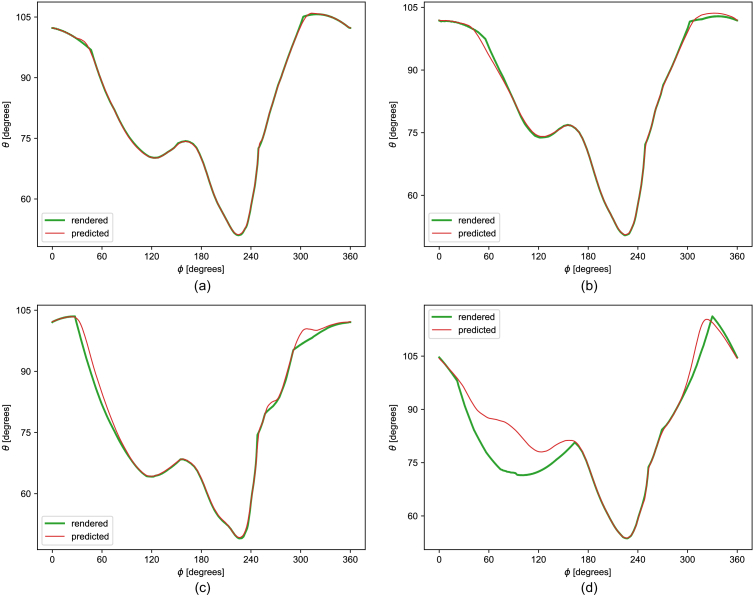
Best, median, 95th percentile, and worst fits of predicted visual field boundaries for random faces. (a) Fit with the lowest error. (b) Fit with the median error. (c) Fit with the 95th percentile error. (d) Fit with the highest error.

Using the optimized values for VF boundaries, we predicted the boundaries for all $N_{random+validation}=10,000$ random heads, and a histogram of their errors is shown in Fig. 8. The lowest error was 0.49, the mean was 6.25, the median was 3.69, and the highest was 334.08. Figure 9 shows the predicted versus rendered VF for four important random faces. We can see that features arising from a combination of multiple parameters cannot be captured effectively by a linear model that considers the parameters individually. In particular, the model is not very good at predicting the sharp corners in the VF boundaries.

## 5. DISCUSSION

This study highlights the importance of considering individual anatomical variations using an *in silico* rendering pipeline when assessing light exposure and VFs. Combining a parametric head shape model and spectral rendering techniques, we developed a novel approach to quantifying how facial anatomy, such as the nose, cheeks, and eyebrows, can impact VF boundaries. The variability in VF boundaries, going up to 18.7% in projected solid angle among 10,000 random heads we considered (Fig. 5), highlights the significant role of head features in shaping individual light exposure. The work demonstrates that predicting VF boundaries for specific individuals is a feasible approach using a combination of 3D face scans and numerical simulations.

This technique can help in creating individualized solutions for lighting design. Since most light loggers measure irradiance, this technique could also help design masks that mimic the VF of individual study participants to be used with light loggers. Spectral rendering techniques can also help statistically assess the impact of VFs in various lighting scenarios.

However, there are some limitations to our approach. The study did not account for other features that might change retinal exposure, including eyelashes [29], ocular structure, pupil size [11,30], squint [10], and eye movements [31]. These additional factors could influence the VF boundary and retinal light exposure, especially in naturalistic lighting situations. We do not expect a significant impact of head position, as gaze direction is more important. The model also does not account for this. We propose a probabilistic convolution-based approach to predict the effect of fixational eye movements. The model only considers symmetric head shapes, and all results shown in this paper are from the perspective of the right eye. However, extending this method to asymmetric faces would not be challenging, as different parameter sets could characterize each eye separately. Using a linear approximation for a fundamentally nonlinear problem introduces potential inaccuracies, as evidenced by the imperfect fits observed for some head shapes. Future work should refine this approach by exploring different methods (e.g., polynomial interpolation or corners/landmarks tracking) for the VF boundary and incorporating calibrated images of real-world scenes or physically realistic renderings to estimate the impact on light exposure. Another opportunity is to calculate individual VFs from 3D face scans or 3D models obtained from 2D photographs [32,33] and compare them with psychophysically measured VFs.

## 6. CONCLUSION

This research provides a foundational rendering-based framework for understanding how head and face anatomy influences the VF and light exposure. We combined 3D modeling and rendering techniques to predict individual VF boundaries from head shapes. Future research should aim to validate these findings empirically in real-world scenarios and explore their application in better estimating personal light exposure.

## Figures and Tables

**Fig. 10.**
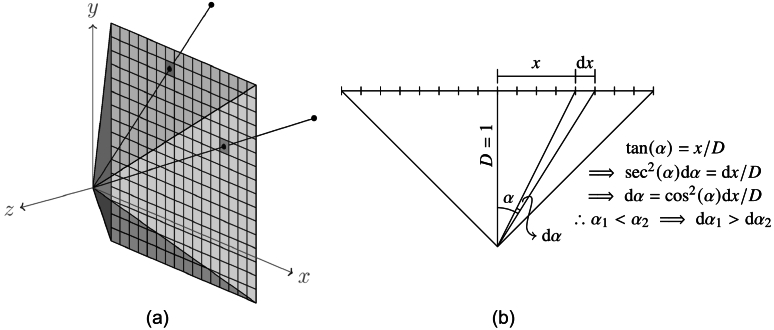
Perspective camera with a flat sensor. (a) An illustration of a perspective camera. (b) Top-down view of the pixels on the x axis.

**Fig. 11.**
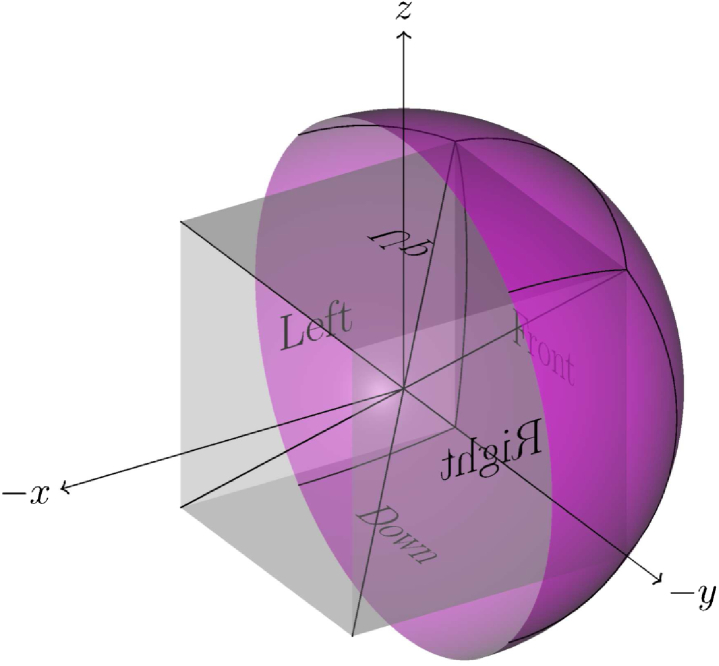
Five cameras cover the hemisphere in front of the eye. The x direction is pointing toward the front.

## Data Availability

Data underlying the results presented in this paper are available in Dataset 1, Ref. [34]. All code used in this work is available under the MIT License on GitHub [20] and archived in Zenodo [21].

## APPENDIX A: PERSPECTIVE CAMERA WITH A FLAT SENSOR

Figure 10(a) illustrates a perspective camera. It involves a center of projection (the eye, shown here at the origin) and a planar sensor. The sensor is perpendicular to the line joining its center and the origin. Points in the scene are mapped to pixels on the sensor by the line segments joining the points to the origin. Figure 10(b) is a top-down view of the pixels on the x axis. It can be seen that the pixels closer to the center of the image subtend larger angles at the origin. If the distance from the origin to the sensor is D , the angle covered by a pixel with width Δ
x at an angle α
 from the center will be cos2⁡
(α
)Δ
x . As the width of all pixels is the same ( =Δ
x ), the ratio of the angles covered by the pixels at the center to those at the edge will be $\cos^{2}(0)/\cos^{2}(FOV/2)=\sec^{2}(FOV/2)$ . If the FOV is 90°, this ratio will be 2.

### A.1. Calculating Irradiance from a Radiance Image

Each pixel in the image subtends a tiny solid angle at the center of the projection. To convert a radiance image to irradiance, we can use this solid angle as a weighting parameter in addition to the cosine factor and sum over all pixels. The solid angle cast at the origin by a rectangle lying on the sensor plane between $\left[x_{min},x_{max},y_{min},y_{max}\right]$ is given by

(A1)
$$solid\;\;angle=\int \frac{projected\;\;area}{distance\;\;squared},$$

(A2)
$$\begin{array}{rl} solid\;\;angle & =\int_{y_{min}}^{y_{max}}\int_{x_{min}}^{x_{max}}\left(\frac{Ddxdy}{{\left(x^{2}+y^{2}+D^{2}\right)}^{1/2}}\right)\\ & \;\times \left(\frac{1}{x^{2}+y^{2}+D^{2}}\right), \end{array}$$

(A3)
$$solid\;\;angle={\left({\left(\tan^{-1}\left(\frac{xy}{D\cdot \sqrt{x^{2}+y^{2}+D^{2}}}\right)\right]}_{x_{min}}^{x_{max}}\right]}_{y_{min}}^{y_{max}}.$$

Let the image have N pixels along both x and y axes. Assuming that the FOV covers the entire image from edge to edge, the dimensions of the pixels Δ
x=Δ
y=(2D/N)tan⁡
(FOV/2) . Hence, the left edges of the pixels will be xmin,i=−
Dtan⁡
(FOV/2)+i⋅
Δ
x , and the right edges will be xmax,i=−
Dtan⁡
(FOV/2)+(i+1)⋅
Δ
x , where i goes from 0 to N−
1 . The same applies to $y_{min,i}$ and $y_{max,i}$ . Using these, we can get the solid angles corresponding to all the pixels and thus convert the radiance image to irradiance.

## APPENDIX B: COVERING THE HEMISPHERE WITH FIVE IMAGES

Because of its geometry, a single perspective camera cannot have an FOV of 180°. Hence, if we need data from the entire hemisphere in front of the eye, we must render multiple images and stitch them together. These images should preferably not have any overlap to avoid over-counting of those regions, and to minimize the difference between the angular coverage of the pixels, the FOV must be as small as possible. Figure 11 shows the setup used in this paper. With each image being a square with an FOV of 90°, we can place five sensors on the faces of a cube to cover the hemisphere in front of the eye. If required, the whole sphere can be covered using six images.

## APPENDIX C: FROM IMAGES TO THE VISUAL FIELD BOUNDARY

Figure 3 shows a typical rendering output. As a constant radiance light source surrounds the head and reflections have been disabled, the values in the output images can only be either 0 (blocked by the facial geometry) or the radiance of the light source ( R , ignoring noise). The pixels along the boundary transitioning from 0 to R lie between those values, and changing the coordinates for these pixels to (θ
,ϕ
) gives us points on the boundary. Breaking the range of ϕ
 values into intervals of 0.01° each, taking an average of all the θ
 values that lie in each of these intervals, we get a smoother function θ
(ϕ
) on the same domain in ϕ
 for all the head shapes. This domain has 36,000 values of ϕ
 going from 0° to 360°. If there is no value of θ
 in one of these intervals, it is linearly interpolated from the neighboring intervals. Periodic boundary conditions are applied at the edges.
